# 5,8-Dibromo-15-cyano-2,11-dithia­[3.3]paracyclo­phane

**DOI:** 10.1107/S1600536811048458

**Published:** 2011-11-23

**Authors:** Hua Zhang, Wenju Liu

**Affiliations:** a Key Laboratory of Pesticide and Chemical Biology of the Ministry of Education, College of Chemistry, Central China Normal University, Wuhan 430079, People’s Republic of China

## Abstract

In the title compound [systematic name: 13,15-dibromo-3,10-dithia­tricyclo­[10.2.2.2^5,8^]octa­deca-1(14),5,7,12,15,17-hexa­ene-6-carbonitrile], C_17_H_13_Br_2_NS_2_, the mean planes of the benzene rings are almost parallel, making a dihedral angle of 1.1 (2)°, and the distance between the ring centroids is 3.294 (3) Å, which is shorter than the normal packing distance of aromatic rings (about 3.4 Å), indicating a strong π–π inter­action. The S atom of one bridging chain is disorderd over two positions with site occupancies of 0.605 (4) and 0.395 (4) for the major and minor components, respectively.

## Related literature

For the preparation of the title compound, see: Wang *et al.* (2006 ▶). For related structures, see: Clément *et al.* (2009 ▶); Jin & Lu (2010 ▶).
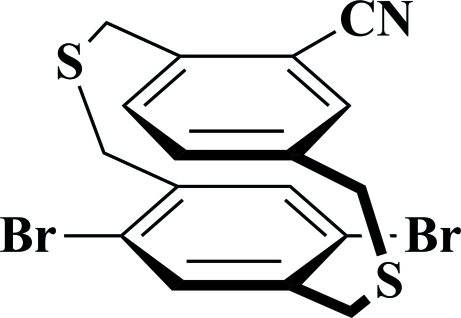


## Experimental

### 

#### Crystal data

C_17_H_13_Br_2_NS_2_
*M* *_r_* = 455.22
Triclinic, 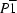

*a* = 6.9433 (11) Å
*b* = 9.0591 (14) Å
*c* = 13.888 (2) Å
α = 79.825 (2)°
β = 85.047 (3)°
γ = 76.275 (2)°
*V* = 834.4 (2) Å^3^
*Z* = 2
Mo *K*α radiation
μ = 5.10 mm^−1^
*T* = 298 K
0.2 × 0.2 × 0.2 mm

#### Data collection

Bruker SMART CCD area-detector diffractometer
5570 measured reflections
3395 independent reflections
2589 reflections with *I* > 2σ(*I*)
*R*_int_ = 0.098

#### Refinement

*R*[*F* ^2^ > 2σ(*F* ^2^)] = 0.059
*wR*(*F* ^2^) = 0.152
*S* = 0.99
3395 reflections
209 parameters
H-atom parameters constrained
Δρ_max_ = 1.39 e Å^−3^
Δρ_min_ = −0.79 e Å^−3^

### 

Data collection: *SMART* (Bruker, 1999 ▶); cell refinement: *SAINT* (Bruker, 1999 ▶); data reduction: *SAINT*; program(s) used to solve structure: *SHELXS97* (Sheldrick, 2008 ▶); program(s) used to refine structure: *SHELXL97* (Sheldrick, 2008 ▶); molecular graphics: *SHELXTL* (Sheldrick, 2008 ▶); software used to prepare material for publication: *SHELXTL* and *PLATON* (Spek, 2009 ▶).

## Supplementary Material

Crystal structure: contains datablock(s) I, global. DOI: 10.1107/S1600536811048458/zq2128sup1.cif

Structure factors: contains datablock(s) I. DOI: 10.1107/S1600536811048458/zq2128Isup2.hkl

Supplementary material file. DOI: 10.1107/S1600536811048458/zq2128Isup3.cml

Additional supplementary materials:  crystallographic information; 3D view; checkCIF report

## Figures and Tables

**Fig. 1.**
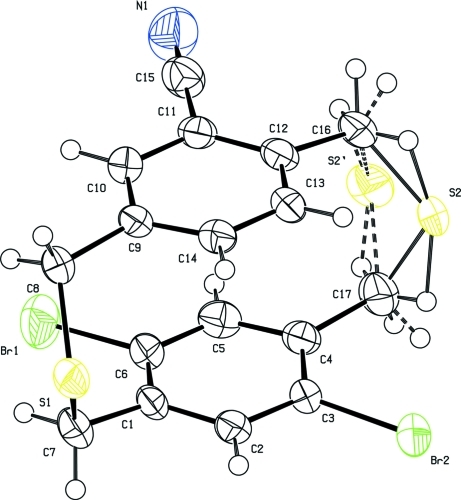
Molecular structure of the title compound showing the atom labeling scheme. Displacement ellipsoids are drawn at the 50% probability level.

## supplementary crystallographic information

### Comment

The benzene dimer of [2,2]paracyclophane is know to play a significant role in chiral catalysis, molecular electronics, and organic solar cells. However, the [3,3]paracyclophane building blocks, which are synthetically more accessible, have received less attention (Clément *et al.*, 2009; Jin & Lu, 2010). Here we report the crystal structure of the title compound, a novel dithia[3,3]paracyclophane bearing cyano and bromido groups.

In the structure of the title compound, C_17_H_13_Br_2_N_1_S_2_, the mean planes of the benzene rings are almost parallel with a dihedral angle of 1.1 (2)° and the distance between the centroids of the rings is 3.294 (3) Å, values obtained by the program *PLATON* (Spek, 2009), which is shorter than the normal packing distance of aromatic rings (about 3.4 Å), indicates a strong π-π interaction. The S atom of one bridging chain is disorderd over two positions with site occupancies of 0.605 (4) and 0.395 (4) for the major and minor components, respectively.

### Experimental

A solution with equimolar amounts of 2,5-dibromo-1,4-bis(mercaptomethyl)benzene (3.26 g, 10 mmol) and 1,4-dibromomethyl-2-cyanobenzene (2.89, 10 mmol) in degassed THF (500 mL) was added dropwise under N_2_ over 12 hours to a refluxing solution of potassium carbonate (6.9 g, 50 mmol) in EtOH (1.5*L*). After additional 2 hours at the reflux temperature (473 K), the mixture was cooled down and the solvent was removed. The resulting residue was treated with CH_2_Cl_2 _(500 mL) and water (500 mL). The organic phase was separated, and the aqueous phase extracted with CH_2_Cl_2_ (three times). The combined organic layers were dried over Na_2_SO_4_, then the solvent was removed, and the resulting solid was chromatographed on silica gel using CH_2_Cl_2_/petroleum ether (1:1, *v*/*v*) as eluent. The product was further purified by recrystallization from toluene (Wang *et al.*, 2006).

### Refinement

All H atoms were positioned with idealized geometry using a riding model, with C—H = 0.93Å for aromatic H atoms, with C—H = 0.97 Å for methylene H atoms, and with *U*_iso_(H) = 1.2*U*_eq_(C). The S atom of one bridging chain is disorderd over two positions with site occupancies of 0.605 (4) and 0.395 (4) for the major and minor components, respectively.

### Crystal data

**Table:**

C_17_H_13_Br_2_NS_2_	*Z* = 2
*M**_r_* = 455.22	*F*(000) = 448
Triclinic, *P*1	*D*_x_ = 1.812 Mg m^−^^3^*D*_m_ = 1.812 Mg m^−^^3^*D*_m_ measured by not measured
Hall symbol: -P 1	Mo *K*α radiation, λ = 0.71073 Å
*a* = 6.9433 (11) Å	Cell parameters from 2268 reflections
*b* = 9.0591 (14) Å	θ = 2.6–26.9°
*c* = 13.888 (2) Å	µ = 5.10 mm^−^^1^
α = 79.825 (2)°	*T* = 298 K
β = 85.047 (3)°	Block, colourless
γ = 76.275 (2)°	0.2 × 0.2 × 0.2 mm
*V* = 834.4 (2) Å^3^	

### Data collection

**Table:**

Bruker SMART CCD area-detector diffractometer	2589 reflections with *I* > 2σ(*I*)
Radiation source: fine-focus sealed tube	*R*_int_ = 0.098
graphite	θ_max_ = 26.5°, θ_min_ = 2.6°
phi and ω scans	*h* = −8→8
5570 measured reflections	*k* = −8→11
3395 independent reflections	*l* = −17→17

### Refinement

**Table:**

Refinement on *F*^2^	Primary atom site location: structure-invariant direct methods
Least-squares matrix: full	Secondary atom site location: difference Fourier map
*R*[*F*^2^ > 2σ(*F*^2^)] = 0.059	Hydrogen site location: inferred from neighbouring sites
*wR*(*F*^2^) = 0.152	H-atom parameters constrained
*S* = 0.99	*w* = 1/[σ^2^(*F*_o_^2^) + (0.0859*P*)^2^] where *P* = (*F*_o_^2^ + 2*F*_c_^2^)/3
3395 reflections	(Δ/σ)_max_ < 0.001
209 parameters	Δρ_max_ = 1.39 e Å^−^^3^
0 restraints	Δρ_min_ = −0.79 e Å^−^^3^

### Special details

**Table:**

Geometry. All e.s.d.'s (except the e.s.d. in the dihedral angle between two l.s. planes) are estimated using the full covariance matrix. The cell e.s.d.'s are taken into account individually in the estimation of e.s.d.'s in distances, angles and torsion angles; correlations between e.s.d.'s in cell parameters are only used when they are defined by crystal symmetry. An approximate (isotropic) treatment of cell e.s.d.'s is used for estimating e.s.d.'s involving l.s. planes.
Refinement. Refinement of *F*^2^ against ALL reflections. The weighted *R*-factor *wR* and goodness of fit *S* are based on *F*^2^, conventional *R*-factors *R* are based on *F*, with *F* set to zero for negative *F*^2^. The threshold expression of *F*^2^ > σ(*F*^2^) is used only for calculating *R*-factors(gt) *etc*. and is not relevant to the choice of reflections for refinement. *R*-factors based on *F*^2^ are statistically about twice as large as those based on *F*, and *R*- factors based on ALL data will be even larger.

### Fractional atomic coordinates and isotropic or equivalent isotropic displacement parameters (Å^2^)

**Table:**

	*x*	*y*	*z*	*U*_iso_*/*U*_eq_	Occ. (<1)
Br1	0.19006 (10)	0.46219 (7)	0.63111 (4)	0.0629 (2)	
Br2	0.19842 (8)	−0.21517 (6)	0.88825 (4)	0.0464 (2)	
C1	0.1424 (6)	0.2546 (5)	0.8092 (3)	0.0361 (10)	
C2	0.1398 (7)	0.1089 (6)	0.8587 (3)	0.0356 (10)	
H2	0.0889	0.0972	0.9230	0.043*	
C3	0.2114 (7)	−0.0199 (5)	0.8146 (3)	0.0339 (10)	
C4	0.2923 (7)	−0.0096 (6)	0.7187 (3)	0.0369 (11)	
C5	0.2780 (7)	0.1387 (6)	0.6673 (4)	0.0415 (11)	
H5	0.3207	0.1511	0.6016	0.050*	
C6	0.2027 (7)	0.2682 (6)	0.7104 (3)	0.0368 (10)	
C7	0.0888 (8)	0.3899 (6)	0.8638 (4)	0.0453 (12)	
H7A	−0.0409	0.3925	0.8965	0.054*	
H7B	0.0795	0.4841	0.8170	0.054*	
C8	0.4903 (8)	0.4041 (6)	0.8808 (4)	0.0415 (11)	
H8A	0.4578	0.4927	0.8296	0.050*	
H8B	0.5828	0.4251	0.9223	0.050*	
C9	0.5938 (6)	0.2654 (5)	0.8335 (3)	0.0348 (10)	
C10	0.6660 (7)	0.2871 (6)	0.7371 (3)	0.0379 (11)	
H10	0.6646	0.3858	0.7037	0.045*	
C11	0.7408 (7)	0.1592 (6)	0.6908 (3)	0.0356 (10)	
C12	0.7430 (7)	0.0109 (6)	0.7372 (3)	0.0364 (10)	
C13	0.6858 (7)	−0.0087 (6)	0.8371 (3)	0.0373 (10)	
H13	0.6966	−0.1074	0.8724	0.045*	
C14	0.6140 (7)	0.1168 (6)	0.8829 (3)	0.0364 (10)	
H14	0.5777	0.1013	0.9493	0.044*	
C15	0.8063 (8)	0.1878 (7)	0.5885 (4)	0.0468 (12)	
C16	0.7960 (8)	−0.1264 (6)	0.6838 (4)	0.0482 (13)	
H16A	0.9294	−0.1836	0.6994	0.058*	0.395 (4)
H16B	0.7961	−0.0895	0.6138	0.058*	0.395 (4)
H16C	0.8168	−0.2192	0.7323	0.058*	0.605 (4)
H16D	0.9207	−0.1252	0.6467	0.058*	0.605 (4)
C17	0.3949 (8)	−0.1478 (7)	0.6717 (4)	0.0516 (14)	
H17A	0.4111	−0.1128	0.6019	0.062*	0.395 (4)
H17B	0.3062	−0.2178	0.6798	0.062*	0.395 (4)
H17C	0.3017	−0.1686	0.6305	0.062*	0.605 (4)
H17D	0.4250	−0.2359	0.7234	0.062*	0.605 (4)
N1	0.8583 (9)	0.2087 (7)	0.5092 (4)	0.0690 (15)	
S1	0.26512 (19)	0.38462 (15)	0.95384 (9)	0.0416 (3)	
S2	0.6293 (3)	−0.2551 (2)	0.71362 (17)	0.0475 (7)	0.605 (4)
S2'	0.6129 (6)	−0.1365 (5)	0.6023 (2)	0.0512 (11)	0.395 (4)

### Atomic displacement parameters (Å^2^)

**Table:**

	*U*^11^	*U*^22^	*U*^33^	*U*^12^	*U*^13^	*U*^23^
Br1	0.0747 (5)	0.0392 (4)	0.0662 (4)	−0.0073 (3)	−0.0055 (3)	0.0078 (3)
Br2	0.0501 (3)	0.0295 (3)	0.0566 (3)	−0.0066 (2)	0.0044 (2)	−0.0060 (2)
C1	0.025 (2)	0.026 (2)	0.056 (3)	0.0008 (18)	−0.0029 (19)	−0.012 (2)
C2	0.030 (2)	0.035 (3)	0.043 (2)	−0.006 (2)	0.0038 (18)	−0.012 (2)
C3	0.030 (2)	0.026 (2)	0.044 (2)	−0.0042 (19)	0.0019 (18)	−0.007 (2)
C4	0.028 (2)	0.039 (3)	0.046 (3)	−0.007 (2)	0.0014 (19)	−0.016 (2)
C5	0.035 (3)	0.048 (3)	0.041 (3)	−0.007 (2)	−0.002 (2)	−0.006 (2)
C6	0.031 (2)	0.030 (2)	0.048 (3)	−0.0033 (19)	−0.0022 (19)	−0.006 (2)
C7	0.040 (3)	0.033 (3)	0.060 (3)	0.001 (2)	0.002 (2)	−0.016 (2)
C8	0.039 (3)	0.029 (3)	0.056 (3)	−0.005 (2)	0.008 (2)	−0.016 (2)
C9	0.027 (2)	0.031 (3)	0.046 (3)	−0.0044 (19)	0.0043 (18)	−0.012 (2)
C10	0.035 (2)	0.031 (3)	0.048 (3)	−0.008 (2)	0.001 (2)	−0.007 (2)
C11	0.027 (2)	0.039 (3)	0.039 (2)	−0.006 (2)	0.0017 (18)	−0.008 (2)
C12	0.026 (2)	0.038 (3)	0.044 (3)	−0.002 (2)	0.0026 (18)	−0.012 (2)
C13	0.032 (2)	0.031 (3)	0.044 (3)	0.000 (2)	0.0033 (19)	−0.005 (2)
C14	0.032 (2)	0.037 (3)	0.038 (2)	−0.006 (2)	0.0046 (18)	−0.006 (2)
C15	0.047 (3)	0.047 (3)	0.044 (3)	−0.005 (3)	0.005 (2)	−0.012 (2)
C16	0.039 (3)	0.040 (3)	0.066 (3)	−0.005 (2)	0.016 (2)	−0.023 (3)
C17	0.050 (3)	0.043 (3)	0.066 (3)	−0.009 (3)	0.007 (3)	−0.027 (3)
N1	0.088 (4)	0.063 (4)	0.055 (3)	−0.021 (3)	0.014 (3)	−0.009 (3)
S1	0.0478 (7)	0.0338 (7)	0.0433 (7)	−0.0065 (6)	0.0111 (5)	−0.0165 (5)
S2	0.0434 (12)	0.0253 (11)	0.0718 (15)	−0.0028 (9)	0.0121 (10)	−0.0163 (10)
S2'	0.056 (2)	0.058 (2)	0.0412 (18)	−0.0094 (18)	0.0122 (15)	−0.0259 (16)

### Geometric parameters (Å, °)

**Table:**

Br1—C6	1.889 (5)	C11—C12	1.381 (7)
Br2—C3	1.899 (5)	C11—C15	1.451 (7)
C1—C2	1.379 (6)	C12—C13	1.401 (7)
C1—C6	1.393 (7)	C12—C16	1.515 (7)
C1—C7	1.510 (7)	C13—C14	1.370 (7)
C2—C3	1.381 (7)	C13—H13	0.9300
C2—H2	0.9300	C14—H14	0.9300
C3—C4	1.397 (6)	C15—N1	1.126 (7)
C4—C5	1.391 (7)	C16—S2'	1.802 (7)
C4—C17	1.512 (7)	C16—S2	1.805 (5)
C5—C6	1.383 (7)	C16—H16A	0.9700
C5—H5	0.9300	C16—H16B	0.9700
C7—S1	1.811 (5)	C16—H16C	0.9700
C7—H7A	0.9700	C16—H16D	0.9700
C7—H7B	0.9700	C17—S2'	1.737 (6)
C8—C9	1.519 (7)	C17—S2	1.774 (6)
C8—S1	1.816 (5)	C17—H17A	0.9700
C8—H8A	0.9700	C17—H17B	0.9700
C8—H8B	0.9700	C17—H17C	0.9700
C9—C14	1.380 (7)	C17—H17D	0.9700
C9—C10	1.385 (6)	S2—H16C	1.4691
C10—C11	1.396 (7)	S2—H17D	1.3851
C10—H10	0.9300		
			
C2—C1—C6	117.4 (4)	C12—C16—S2'	115.1 (4)
C2—C1—C7	119.6 (4)	C12—C16—S2	113.9 (4)
C6—C1—C7	123.0 (4)	S2'—C16—S2	56.8 (2)
C1—C2—C3	121.3 (4)	C12—C16—H16A	108.8
C1—C2—H2	119.3	S2'—C16—H16A	135.9
C3—C2—H2	119.3	S2—C16—H16A	108.8
C2—C3—C4	121.9 (4)	C12—C16—H16B	108.8
C2—C3—Br2	118.2 (3)	S2'—C16—H16B	54.2
C4—C3—Br2	119.9 (4)	S2—C16—H16B	108.8
C5—C4—C3	115.8 (4)	H16A—C16—H16B	107.7
C5—C4—C17	120.5 (4)	C12—C16—H16C	108.1
C3—C4—C17	123.7 (5)	S2'—C16—H16C	108.4
C6—C5—C4	122.3 (4)	S2—C16—H16C	54.4
C6—C5—H5	118.8	H16A—C16—H16C	59.6
C4—C5—H5	118.8	H16B—C16—H16C	143.1
C5—C6—C1	120.6 (4)	C12—C16—H16D	108.8
C5—C6—Br1	117.6 (4)	S2'—C16—H16D	108.7
C1—C6—Br1	121.7 (4)	S2—C16—H16D	137.0
C1—C7—S1	113.7 (4)	H16A—C16—H16D	50.1
C1—C7—H7A	108.8	H16B—C16—H16D	60.1
S1—C7—H7A	108.8	H16C—C16—H16D	107.4
C1—C7—H7B	108.8	C4—C17—S2'	117.2 (4)
S1—C7—H7B	108.8	C4—C17—S2	118.4 (4)
H7A—C7—H7B	107.7	S2'—C17—S2	58.5 (2)
C9—C8—S1	115.4 (3)	C4—C17—H17A	107.7
C9—C8—H8A	108.4	S2'—C17—H17A	51.6
S1—C8—H8A	108.4	S2—C17—H17A	107.7
C9—C8—H8B	108.4	C4—C17—H17B	107.7
S1—C8—H8B	108.4	S2'—C17—H17B	134.1
H8A—C8—H8B	107.5	S2—C17—H17B	107.7
C14—C9—C10	118.5 (4)	H17A—C17—H17B	107.1
C14—C9—C8	121.9 (4)	C4—C17—H17C	108.2
C10—C9—C8	119.6 (4)	S2'—C17—H17C	108.3
C9—C10—C11	119.2 (4)	S2—C17—H17C	132.5
C9—C10—H10	120.4	H17A—C17—H17C	63.7
C11—C10—H10	120.4	H17B—C17—H17C	45.3
C12—C11—C10	122.2 (4)	C4—C17—H17D	107.8
C12—C11—C15	120.3 (5)	S2'—C17—H17D	107.7
C10—C11—C15	117.3 (4)	S2—C17—H17D	50.9
C11—C12—C13	117.2 (4)	H17A—C17—H17D	144.4
C11—C12—C16	122.7 (4)	H17B—C17—H17D	64.1
C13—C12—C16	120.1 (4)	H17C—C17—H17D	107.1
C14—C13—C12	120.3 (4)	C7—S1—C8	103.7 (2)
C14—C13—H13	119.8	C17—S2—C16	106.1 (3)
C12—C13—H13	119.8	C17—S2—H16C	135.9
C13—C14—C9	122.0 (4)	C16—S2—H17D	134.8
C13—C14—H14	119.0	H16C—S2—H17D	152.9
C9—C14—H14	119.0	C17—S2'—C16	107.8 (3)
N1—C15—C11	179.4 (7)		
			
C6—C1—C2—C3	5.8 (7)	C10—C11—C12—C16	171.0 (5)
C7—C1—C2—C3	−171.8 (4)	C15—C11—C12—C16	−5.2 (7)
C1—C2—C3—C4	0.9 (7)	C11—C12—C13—C14	5.8 (7)
C1—C2—C3—Br2	−179.2 (3)	C16—C12—C13—C14	−171.8 (5)
C2—C3—C4—C5	−6.2 (7)	C12—C13—C14—C9	0.5 (7)
Br2—C3—C4—C5	174.0 (3)	C10—C9—C14—C13	−6.2 (7)
C2—C3—C4—C17	172.2 (5)	C8—C9—C14—C13	172.1 (5)
Br2—C3—C4—C17	−7.6 (6)	C11—C12—C16—S2'	−71.3 (6)
C3—C4—C5—C6	4.9 (7)	C13—C12—C16—S2'	106.1 (5)
C17—C4—C5—C6	−173.7 (5)	C11—C12—C16—S2	−134.3 (4)
C4—C5—C6—C1	1.8 (7)	C13—C12—C16—S2	43.1 (6)
C4—C5—C6—Br1	−179.4 (3)	C5—C4—C17—S2'	41.4 (7)
C2—C1—C6—C5	−7.2 (7)	C3—C4—C17—S2'	−137.1 (5)
C7—C1—C6—C5	170.4 (5)	C5—C4—C17—S2	108.4 (5)
C2—C1—C6—Br1	174.1 (3)	C3—C4—C17—S2	−70.0 (6)
C7—C1—C6—Br1	−8.4 (6)	C1—C7—S1—C8	65.3 (4)
C2—C1—C7—S1	66.7 (5)	C9—C8—S1—C7	−70.7 (4)
C6—C1—C7—S1	−110.8 (5)	C4—C17—S2—C16	−64.2 (5)
S1—C8—C9—C14	−41.7 (6)	S2'—C17—S2—C16	42.0 (3)
S1—C8—C9—C10	136.6 (4)	C12—C16—S2—C17	64.3 (5)
C14—C9—C10—C11	5.4 (7)	S2'—C16—S2—C17	−41.1 (3)
C8—C9—C10—C11	−172.9 (4)	C4—C17—S2'—C16	65.6 (5)
C9—C10—C11—C12	0.9 (7)	S2—C17—S2'—C16	−42.6 (3)
C9—C10—C11—C15	177.2 (4)	C12—C16—S2'—C17	−60.5 (5)
C10—C11—C12—C13	−6.5 (7)	S2—C16—S2'—C17	42.6 (3)
C15—C11—C12—C13	177.3 (4)		
